# ROS and Abiotic Stress in Plants 2.0

**DOI:** 10.3390/ijms241511917

**Published:** 2023-07-25

**Authors:** Veselin Petrov, Tsanko Gechev

**Affiliations:** 1 Center of Plant Systems Biology and Biotechnology, 139 Ruski Blvd., 4000 Plovdiv, Bulgaria; gechev@cpsbb.eu; 2 Department of Plant Physiology, Biochemistry and Genetics, Agricultural University Plovdiv, 12 Mendeleev Str., 4000 Plovdiv, Bulgaria; 3 Department of Plant Physiology and Molecular Biology, University of Plovdiv, 24 Tsar Assen Str., 4000 Plovdiv, Bulgaria

Climate insecurity and extreme weather events have stimulated efforts to enhance plant resilience and productivity in adverse environmental conditions. Given the significance of reactive oxygen species (ROS) and oxidative stress in plant fitness and performance, it is not surprising that a major goal of the plant research community is to elucidate the mechanisms by which plants regulate ROS levels, maintain the redox balance and respond to stressors. The progress in this direction is facilitated by the advancements in genetics, molecular and systems biology, which provide tools to generate large amounts of data and effectively verify it. This trend is expected to continue in the future. Taking this into consideration and following the success of the first Special Issue dedicated to “ROS and Abiotic Stress in Plants” [1], it was decided to prepare a second Special Issue on the same topic. The current collection, “ROS and Abiotic Stress in Plants 2.0”, gathers ten new publications, among which seven are experimental studies and three are review papers. In this editorial, we provide a brief summary of their main results and conclusions, including important aspects of the correlation between ROS homeostasis, diverse abiotic stressors, and their implications for crop yield.

Two of the experimental studies in this Special Issue are devoted to enzymatic components of the ROS scavenging systems in plants. Eljebbawi et al. (2023) investigated the expression of plant-specific class III peroxidases (CIII *Prxs*) in the roots of Pyrenean populations of *Arabidopsis thaliana*, with the aim being to reveal more details on their functional plasticity in response to various abiotic stressors [2]. For this purpose, they selected the studied populations carefully, so that they had a close geographic distribution, and thus the same origin, but were subjected to quite dissimilar environments as a result of an altitudinal gradient. Initially, three tolerant populations were identified, based on their better root growth under different stress conditions, namely cold, heat and salinity. The subsequent RNA-seq analysis of CIII *Prxs* was followed by treatment- and genotype-based differential expression (DE) comparisons. These allowed the discrimination of both the natural variation between the populations themselves, as well as the specific gene modulation by the particular stresses. As a result, many Arabidopsis CIII *Prxs* were found to be associated with cold, heat, salinity or their combinations. The list of cold-responsive CIII *Prxs* amounted to 19 entries, of which 9 were regulated only by low temperature (*Prx16*, *Prx24*, *Prx27*, *Prx47*, *Prx55*, *Prx57*, *Prx64*, *Prx66* and *Prx70*); for heat, the total number of genes was 17, with 7 specific only for this condition (*Prx17*, *Prx39*, *Prx53*, *Prx58*, *Prx61*, *Prx65*, and *Prx67*); and for salinity, the list contained 17 genes, with 6 specific ones (*Prx14*, *Prx15*, *Prx40*, *Prx49*, *Prx52* and *Prx62*). Finally, three of the *Prxs* (*Prx8*, *Prx71* and *Prx73*) appeared to be broadly sensitive to unfavourable environments, since they were upregulated under all applied stresses.

In turn, the paper by Li et al. [3] is devoted to the investigation of the importance of chloroplast thylakoidal ascorbate peroxidase (tAPX) for the oxidative stress tolerance in the woody species *Populus tormentosa*. They used an approach utilizing both an overexpression mutant (OX-PtotAPX) and an antisense one (anti-PtotAPX), whose morphology and responses were compared with wild type plants with and without application of the oxidative burst inducer methyl viologen (MV), also known as paraquat. The team monitored the observable phenotypes, the subcellular structure in leaves and stems, photosynthetic parameters like Fv/Fm, vascular tissue morphology, and various parameters related to the redox status and the state of the antioxidant systems. Their experiments demonstrated that under normal conditions, the mutants did not significantly differ from the wild genotype. This suggests that in the absence of stress, the other ROS scavenging enzymes, for example the cytoplasmic APX, can compensate for the loss of tAPX activity in anti-PtotAPX plants. However, when oxidative stress was triggered, growth and developmental processes were differentially affected. Overall, the overexpressor appeared to be more tolerant to the effects of MV than the controls, while the antisense mutant displayed the opposite phenotype and had more expressed stress symptoms. Therefore, tAPX emerges as a crucial antioxidant enzyme in the chloroplasts of *P. tormentosa*, which is necessary for effective H_2_O_2_ detoxification under oxidative stress burden. The authors propose that it can serve as a genetic resource for modulating the stress tolerance of woody species.

Three other papers in this Special Issue deal with elements of the transcriptional regulatory networks involved in specific stress responses. Yao et al. [4] studied the MYB transctiption family (TF) members in the Chinese crab apple *Malus baccata* (L.) and selected *MbMYB4*, which was highly responsive to cold and drought treatments in seedlings, for further analysis. The gene was cloned and overexpressed in *A. thaliana*. Of the six initially obtained T_2_ transformed lines, the three that had a higher expression of *MbMYB4* were characterized in more detail by monitoring the phenotype and assessing the chlorophyll and proline contents, the activities of catalase and peroxidase, the expression of marker genes as well as the conductivity and lipid peroxidation states under cold and drought conditions. The observations showed that the overexpression of *MbMYB4* successfully conferred significantly enhanced tolerance in *A. thaliana* to both stress treatments. The authors also included a model of the pathways in which *MbMYB4* participates during cold and drought. According to this model, low temperatures cause *MbMYB4* to bind to the promoter of the CBF transcription factor, which activates the CBF1 and CBF3 effectors, and they in turn regulate downstream cold-responsive genes like *COR15a* and *RD29a*. In the case of drought, *MbMYB4* interacts with ABA and modulates the transcription of signal transduction genes like *NCED3* and *SnRK2.4*.

The paper by Zheng et al. [5] deals with the *Nuclear Factor-Y* (*NF-Y*) gene family of transcription factors and their role in nutrient deficiencies in rapeseed (*Brassica napus*). Initially, the physiological responses of this important crop were monitored in conditions of limited nitrogen, phosphorus and potassium. The plants appeared to be the most sensitive to N deprivation and less so to K shortage. Then, the authors identified 108 *NF-Y* genes in *B. napus*, performed a detailed study on their gene structure, chromosomal location, phylogenetic relationships, conserved motifs, evolutionary patterns and synteny, and subsequently investigated their potential contribution to nutrient starvation responses. The analyses revealed that the distribution of the 108 genes was 38 in the NF-YA, 46 in the NF-YB and 24 in the NF-YC subfamilies, respectively, and their expansion in rapeseed was caused mainly by segmental duplication events. Moreover, evidence for purifying selective pressure of the *NF-Y* family members was presented. As demonstrated by RNA-seq, in conditions of nutrient depletion for N, P and K, a large part of the *NF-Y* genes, mostly from the NF-YA subfamily, were differentially expressed, with 34 regulated in response only to a single element deficit. Finally, based on their coexpression relationships, 16 “hub” genes were selected, and their expression was additionally verified in five different tissues under N deficiency. These were proposed as good candidates for future functional studies.

Since they are potent transcriptional regulators, microRNAs (miRNAs) have been shown to play important roles in plant responses against abiotic stresses. The team of Liu et al. [6] focused on the functions of a miRNA from the Chinese woody plant *Caragana korshinskii*, which is used as a feed crop and is characterized as having significant drought tolerance. In one of their previous studies, they had found that miR2119 is one of the responsive miRNAs to drought stress in *C. korshinskii* and predicted that its target gene is *Bax Inhibitor-1* (*BI-1*), encoding a key protein for the maintenance of endoplasmic reticulum homeostasis. In the current report, they continued their research on CkmiR2119 and firstly validated its interaction with *CkBI-1*. Expression analysis of both factors in leaves and stems under drought conditions demonstrated their opposite trends of accumulation, which supports their relationship. Moreover, the ability of CkmiR2119 to cleave *CkBI-1* and reduce its expression level was directly demonstrated by GUS assays in tobacco leaves that transiently expressed CkmiR2119 and *CkBI-1*, individually and in combination. Next, evidence was shown that the mechanism through which *CkBI-1* and its regulator CkmiR2119 can influence the drought responses in this species involve programmed cell death (PCD)—*CkBI-1* can inhibit the progress of PCD, while CkmiR2119 can relieve this effect by mediating the degradation of *CkBI-1*. This is consistent also with the behavior of CkmiR2119 and *CkBI-1* in the two different organs. In stems, CkmiR2119 is upregulated during the drought treatment, with a concomitant decrease in *CkBI-1* transcription, which eventually promotes the better development of xylem elements through PCD, ultimately resulting in increased efficiency of water transport. In contrast, in leaves under drought conditions, the miRNA is depleted and *CkBI-1* accumulates, resulting in a reduced PCD burden and lower withering.

Another of the published manuscripts focuses on the comparison of the transcriptional responses of two closely related species, *Arabidopsis thaliana* and *Pachycladon cheesemanii*, to low temperature, salinity stress and UV-B radiation [7]. Unlike *A. thaliana*, *P. cheesemanii* demonstrates significant tolerance to harsh conditions. Therefore, this study was designed with the aim to uncover shared and unique molecular mechanisms that contribute to this important trait, as well as specific responses depending on the type of stress within both species. The results obtained indicate that cold stress induced some common genes involved in trehalose, phenylpropanoid and oxylipin metabolism, and circadian rhythm-related processes in the two plants. On the other hand, only in *A. thaliana*, low temperatures also led to the upregulation of genes participating in low-molecular-weight carbohydrates, while in *P. cheesemanii*, the specific response included some plant hormone pathways and glucosinolate metabolism. In turn, salt treatment triggered relatively generic GO terms in both species, stress-induced wax biosynthesis associated with the cuticle development in Arabidopsis only, and tetrapyrrole and proline catabolic processes in *P. cheesemanii*. Finally, UV-B radiation resulted in the common modulation of pathways involved in the metabolism of L-ascorbic acid, L-phenylalanine, and chorismate. In addition, this condition impacted callose-related cell wall defense specifically in Arabidopsis, and anthocyanin biosynthesis in *P. cheesemanii*. The authors conclude their work by pointing out a useful direction for future studies on the impressive abiotic stress tolerance of *P. cheesemanii*, which includes investigating the possible contribution of its polyploid genome.

The last of the experimental papers in this Special Issue is dedicated to one very peculiar and intriguing type of stress—that induced by spaceflight. Zeng et al. [8] investigated the transgenerational effects of spaceflight on an F2 rice progeny by evaluating some agronomic traits, assessing the redox state and carrying out two types of -omics analyses—proteomics and metabolomics. The study demonstrates that while seed germination and plant height at two stages of development, namely three-leaf and tillering stages, were not affected by spaceflight, the number of tillers were slightly reduced. However, a number of parameters related to ROS homeostasis and oxidative stress were considerably different, providing evidence for persistent stress memory and inherited disruption of the ROS balance. These parameters include elevated H_2_O_2_ concentration, lipid peroxidation, electrolyte leakage and activities of some antioxidant enzymes like catalase, peroxidases and superoxide dismutase in the spaceflight group compared to the controls. The subsequent -omics experiments revealed a concomitant reorganization of the proteome and metabolome. At the proteome level, the F2 rice progeny had some altered signal transduction and stress response pathways, as well as increased genome instability and reduced protein synthesis, modification and turnover rate. At the metabolome level, the spaceflight had drastically influenced both primary metabolites like amino acids, sugars, vitamins and cofactors, and secondary metabolites like flavonoids. Overall, this interesting report provides solid data demonstrating that spaceflight and the stressors associated with it, like altered gravity, space radiation, and magnetic fields, exert in plants persistent biological effects that transgress across generations and leave an imprint on the molecular state of the offspring. This should be taken into consideration in future space-related studies and programmes.

The review papers added in this Special Issue discuss signals from three different origins that influence and interact with the abiotic stress responses in plants. The first one is dedicated to the crosstalk between ROS and the important gaseous signaling molecule nitric oxide (NO) [9]. The authors note that similar to ROS, NO is also known to participate in numerous signal transduction processes and responses to various stimuli including stresses. Moreover, exogenous application of NO donors has been shown to positively affect plant abiotic stress tolerance. This review provides specific details on the involvement of NO in three types of abiotic conditions—drought, salinity and increased concentrations of heavy metals. For additional reference, lists of recent research studies devoted to NO and each of the stresses are also supplied. The association of NO with drought is presented in several layers, namely NO’s effects mediated through interactions with the antioxidant system, the role of NO in the ABA-dependent stomatal closure and NO’s influence on drought-responsive genes. In turn, the section on metal stress is subdivided into sections covering cadmium, copper, arsenic, zinc, lead, chromium and mercury. In the end, the paper contains a useful recommendation for future study directions in the field of NO research. It is suggested that the effects of various different NO donors on plants, including NO in its gaseous state, should be taken into consideration and investigated by comparison and simultaneous application.

The second review provides a systemic summary of the role of the phytohormone salicylic acid in multiple abiotic stresses in rice [10]. Development of strategies to improve abiotic stress tolerance of rice is crucial for a significant portion of the world, encompassing some of the most populous countries, where this is a main source of calories. In this context, knowledge on SA will be instrumental, due to the power that this has molecule to regulate a plethora of processes, including resilience to abiotic and biotic stresses. The manuscript elaborates consecutively on SA’s influence on salinity, drought, high and low temperatures, metal toxicity and nutrient deficiency, all of which are finally summed up in a schematic representation. Next, the mechanisms through which SA can stimulate stress tolerance are presented in detail in sections on the interaction of SA with osmolytes, its role in mineral acquisition, its modulation of ROS-signaling and antioxidant activities, its interference with secondary metabolism and its crosstalk with other hormones. The paper culminates with a paragraph on the future perspectives and a list of possible SA-related issues to be further investigated in rice.

Finally, Berrios and Rentsch [11] offer an intriguing overview of the links that exist between the maintenance of ROS homeostasis and plant-associated microbiomes. After introducing the concept of their manuscript, the authors delve into the specificities of ROS signaling in plant development and cover topics like seed germination and the breaking of dormancy, root elongation, shoot meristem growth and maintenance, cell differentiation and floral organs maturation. Some aspects of the roles of ROS in both abiotic and biotic stresses are discussed as well. Then, the important relationships and interactions between the ROS levels in plants and the plant microbiome are presented, with details on the effects of the different microbial populations, the influence of both spatial and temporal factors on this complex system, and most importantly, the potential to exploit the plant–microbial interactions for reducing the ROS burden, with obvious practical applications to stimulate crop yield. Still, it is made clear that in order to fully understand the multi-faceted and intricate plant–plant, plant–microbe and microbe–microbe interplay in the soil environment, many challenges remain and new approaches, preferably holistic ones, are required.

Ensuring global food security is of utmost importance to achieve sustainability in the era of climate change, as the negative impacts on food production and distribution have the potential to exacerbate hunger and malnutrition, particularly in vulnerable populations. Therefore, the research on plant responses to various stresses, which ultimately affect their yield, has not only fundamental but also major practical value. However, the intrinsic complexity of these responses, determined by multilayer regulation, dozens of players, crosstalk with other growth and development processes, and adoption of specific strategies by various taxa and even species, poses significant challenges and slows down the achievement of breakthrough in this field. At present, the devising of a universally valid approach to enhance crop tolerance to unfavourable conditions still seems like a far-off objective, but measurable results for particular species or taxa and environmental contexts are reached with gradual steady steps.

The research papers included in this Special Issue add relevant pieces of the puzzle. They expand our knowledge on components of the antioxidant defenses like peroxidases and ascorbate peroxidase [2,3], transcriptional regulators from different plants that exert roles in the responses to various stresses [4,5,6], common and specific responses to various abiotic stress stimuli in a susceptible and tolerant plant [7], and even the transgenerational pervasiveness of stress memory induced by spaceflight [8]. Additionally, the three published review papers provide comprehensive summaries on the current state-of-the art of our understanding of the interference of ROS homeostasis and other signals [9,10,11]. It is our hope that they will serve as a source of motivation for valuable future research endeavours.
